# Energetics of Ortho-7 (Oxime Drug) Translocation through the Active-Site Gorge of Tabun Conjugated Acetylcholinesterase

**DOI:** 10.1371/journal.pone.0040188

**Published:** 2012-07-11

**Authors:** Vivek Sinha, Bishwajit Ganguly, Tusar Bandyopadhyay

**Affiliations:** 1 Indian Institute of Science Education and Research Kolkata, Mohanpur Campus, Mohanpur, Nadia, India; 2 Analytical Science Discipline, Central Salt & Marine Chemical Research Institute (Council of Scientific and Industrial Research), Bhavnagar, Gujarat, India; 3 Theoretical Chemistry Section, Chemistry Group, Bhabha Atomic Research Centre, Trombay, Mumbai, India; University of Illinois, United States of America

## Abstract

Oxime drugs translocate through the 20 Å active-site gorge of acetylcholinesterase in order to liberate the enzyme from organophosphorus compounds’ (such as tabun) conjugation. Here we report bidirectional steered molecular dynamics simulations of oxime drug (Ortho-7) translocation through the gorge of tabun intoxicated enzyme, in which time dependent external forces accelerate the translocation event. The simulations reveal the participation of drug-enzyme hydrogen bonding, hydrophobic interactions and water bridges between them. Employing nonequilibrium theorems that recovers the free energy from irreversible work done, we reconstruct potential of mean force along the translocation pathway such that the desired quantity represents an unperturbed system. The potential locates the binding sites and barriers for the drug to translocate inside the gorge. Configurational entropic contribution of the protein-drug binding entity and the role of solvent translational mobility in the binding energetics is further assessed.

## Introduction

Acetylcholinesterase (AChE), a serine hydrolase that belongs to esterase family in higher eukaryotes, is responsible for the termination of the nerve impulse at cholinergic synapses. The enzyme’s catalytic efficiency towards removal of cationic neurotransmitter, acetylcholine is one of the fastest and close to diffusion controlled limit [1]. Because of the pivotal role that AChE plays, the active-site of the enzyme, located deep inside a 20 Å gorge [2] is an attractive target for the design of inhibitors. Clinically a moderate inhibition of AChE is desirable for the treatment of a number of nerve diseases [2]–[5]. However, prolonged inhibition of AChE, particularly by covalent binding to the active-site serine, is invariably lethal. One such lethal action is caused by highly toxic organophosphorus (OP)-type chemical warfare agents (nerve agents) [6] such as tabun. The medical management of nerve agent intoxication includes the use of nucleophiles, such as oximes that can reactivate the enzyme [7].

**Figure 1:**
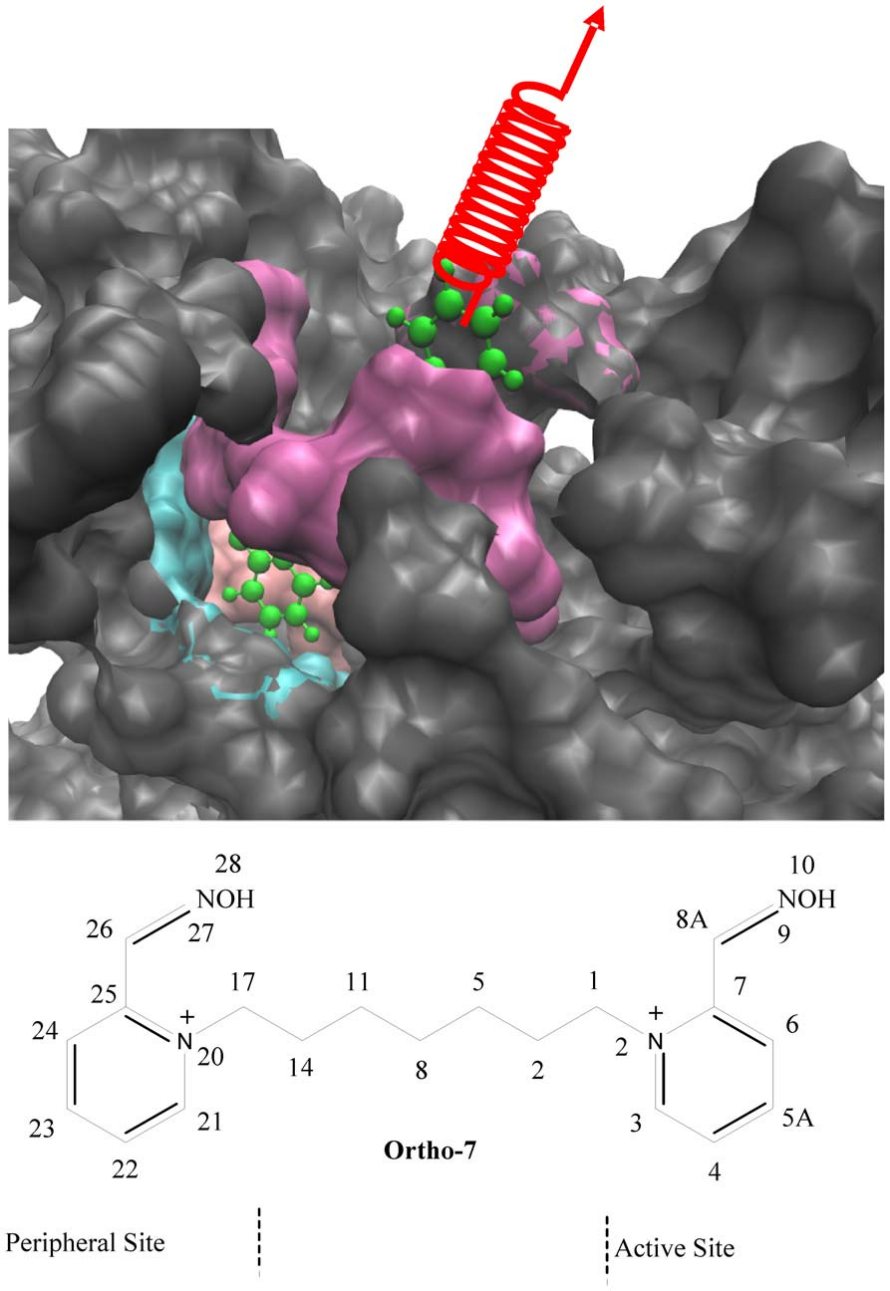
A section of the mAChE gorge is shown in the surface representation at the top. The catalytic triad and the anionic subsite is highlighted in pink and cyan, respectively while the PAS region is presented in mauve. The drug molecule, Ortho-7 (green) is pulled with a harmonic spring from the protein gorge as shown. Bottom shows the chemical structure of Ortho-7 [1,7-heptylene-bis-N,N’-2-pyridiniumaldoxime]. The heavy atoms are numbered for referral purpose. One of the two pyridinium rings (active pyridinium) is directed towards the active triad region while the other (peripheral pyridinium) towards the PAS region of the AChE enzyme.

In Mus musculus AChE (mAChE), the base of the gorge consists of two subsites: the esteratic subsite comprising the active catalytic triad (S203, E334, and H447) and an anionic subsite (made up of W86, E202, Y337) [8]. In the third domain, a hydrophobic region is contiguous with or near the loci of the previous two domains and is responsible for binding aryl substrate and active-site ligands. A fourth domain, on the other hand, is over 20 Å away from the active-site, located at the rim of the gorge and has been called peripheral anionic site (PAS) [9], [10], and comprises of aromatic residues Y72, Y124, W286 and Y341 and an anionic residue D74. The four domains together produce an effective binding mechanism with ligand: oxime drugs intended to repair the OP reacted enzyme first have to be captured from the environment by the PAS and then have to push part several hydrophobic aromatic residues lining the gorge wall that are too bulky to allow it a free passage to the active site. The complex kinetics of AChE reactivation depend both on the chemical structure of the OP conjugate of the enzyme and the oximes [11]–[14], which often lacks a clear structure-activity relationship between them. Among the OP intoxicants, liberating the free enzyme from tabun intoxication is the most difficult as the most known oxime reactivators are ineffective, while the best known reactivator, Ortho-7 (see Fig. 1) is only 24% efficient [10].

Recently, through a novel combination of mass spectrometric and crystallographic measurements, the structure of tabun conjugated mAChE in complex with Ortho-7 is obtained [10]. It has been found that one of the two pyridinium rings of Ortho-7 binds reversibly with the PAS (peripheral pyridinium) and the other (active pyridinium) is directed towards the active-site of the enzyme through the linker. Both the pyridinium rings of the drug molecule experiences strong cation-π interactions with aromatic residues; peripheral pyridinium ring with the PAS residues Y72 and W286, and the active pyridinium ring with the W86 and Y337 residues. The active pyridinium ring of the drug is found to be too bulky to be fit in the active site. In addition, the oxime oxygen is found to be closest to the phosphor atom of the tabun conjugate, a prerequisite of reactivation, when the torsion between oxime group and the active pyridinium ring is 60? [10]. However, this configuration adopts a high potential energy. These structural evidences explain the poor reactivation efficiency of Ortho-7. Ligand translocation through the active-site gorge is of course a subtle issue. For example, the dynamics of passage of bispyridinium oxime drugs through the active-site gorge and the issue of complementarities of the two pyridinium rings at PAS and in the active site of the tabun free mAChE has been recently explored through molecular dynamics (MD) simulations [15].

Binding and unbinding events of ligands happen on the milliseconds or longer time scale [16], [17] and is therefore not amenable to study through conventional MD (CMD) simulation techniques. As a result, accelerated MD protocols of the likes of steered MD (SMD) [15], [18], [19] and metadynamics [20] have been attempted to elucidate the mechanism of passage of ligands through the active-site gorge of AChE. The SMD results of inhibitors [18], [19] and oxime drugs [15] clearly depicts the participation of ligand-protein direct hydrogen bonds (DHB), hydrophobic interactions (HI), water bridges (WB) connecting the ligand with the protein. These interactions either facilitates or hinder the passage of the ligands to the active triad. The metadynamics simulation work on cationic ligand further unravel the pivotal role of PAS in the uptake of the ligand through cation- π interaction and deduce the potential of mean force (PMF) of ligand penetration into AChE gorge as a function of suitably chosen reaction coordinate [20]. Earlier to this, successful attempt was made to evaluate the similar PMF of ligand penetration by umbrella sampling [21]. Both the nonequilibrium [20] and equilibrium [21] MD techniques clearly identifies the barrier and traps, the key residues of AChE and their interactions that orchestrate the ligand passage along the conduction pathway.

The present work seeks to investigate translocation events of the oxime drug, Ortho-7 through the active-site gorge of tabun conjugated mAChE. To accelerate the translocation which is otherwise too sluggish to be contemplated by CMD, we have adopted the techniques of SMD in which an external force is applied to the drug molecule. In order to concisely characterize the conduction process and the protein-drug physical interactions thereby, we utilize forward-reverse bidirectional SMD protocol [22]–[24] and construct the PMF, namely the free energy profile along the translocation pathway.

Nonequilibrium work theorems such as Jarzynski’s equality [25] and Crooks fluctuation theorem [26] have been exploited to find the free energy differences between two states. The nonequilibrium work theorems connect an equilibrium quantity (free energy) with measured nonequilibrium work, however, it does so as a function of time and not as a function of fluctuating molecular positions. And hence PMFs cannot be directly measured utilizing them. The algorithm, as proposed by Hummer and Szabo [27], [28] (HS) could circumvent this problem through repeated unidirectional nonequilibrium work measurements while applying Jarzynski’s equality theorem. However, the obtained results suffers from the issues of convergence. Bidirectional SMD simulations, on the other hand, scores over Jarzynski equality based unidirectional SMD protocol. This is because the rare trajectories that one misses to count for in the forward simulation can be frequently encountered during the reverse steps and vice versa. As a result, the convergence criteria of the measured free energy can be easily met. Following this, here we adopt recently proposed PMF estimators [22], [23] that essentially utilizes HS [27], [28] reweighting technique and Bennett acceptance ratio (BAR) [29] method and obtain the unperturbed PMF as a function of protein-drug centre of mass (COM) separation for the oxime drug translocation through the tabun conjugated mAChE gorge via bidirectional SMD simulations.

## Methods

We have performed all-atom MD simulations of tabun conjugated protein molecule complexed with Ortho-7. In order to recover the PMF, repeated SMD runs of binding and unbinding transitions are performed by attaching a harmonic spring to the COM of the drug molecule that moves with a constant velocity.

### 1. Modeling

The modeling of the fully hydrated protein-drug complex was carried out using the MD simulation program GROMACS 3.3.2 [30] with GROMOS 96 [31] force field parameter sets. The structure of the complex, mAChE-Tabun•Ortho-7 (PDB ID 2JF0) [10] was repaired for missing residues and missing heavy atoms [15]. Out of the dimeric X-ray crystal structure of the complex obtained at 2.5 Å resolution, only the monomer A chosen for modeling, since monomer A is better resolved in the electron density map. OP compound, tabun upon conjugation transforms the active S203 residue of mAChE into a phosphorous conjugate named as SUN [10]. The geometry of the oxime drug and as well of SUN residue was optimized with HF/6–31 G** basis set and their partial atomic charges were determined by the CHelpG algorithm as implemented in Gaussian 03 program [32]. While the topology and force field bonded parameters of the drugs were determined by the PRODRG 2.5 beta server [33] those of SUN residue were evaluated from Gaussian 03 program using the optimized structure and using the keyword frequency. The obtained bonded parameters of SUN was transcribed to GROMOS 96 format and the non bonded parameters were ascribed to the existing GROMOS96 parameter set. The force field parameters of SUN are listed in the Supporting Information (File S1). Crystallographically resolved drug molecule located in the active-site of tabun conjugated complex was removed and the resulting protein molecule was equilibrated for 5 ns at NPT ensemble through Berendsen temperature and pressure coupling schemes [34]. Time dependent rms deviations of the Cα atoms of the equilibrated structures of the tabun conjugated protein molecule from the crystal structure was found to lie within 2 Å. The modeling of the tabun conjugated serine residue, SUN is thus appears to be justified.

The drug molecule was then replaced in the active-site following the crystallographically determined equilibrium position and the system was solvated with simple point charge water molecules. Required number of positive (Na^+^) and negative (Cl^−^) ions were added to maintain electroneutrality. The resulting system was comprising of more than 100,000 atoms. The solvent and the solute were then separately coupled to temperature reservoir at 300 K. The solvent was first heated up to 300 K for 300ps with protein and drug molecule fixed and subsequently the solutes were heated up to 300 K for 200 ps with Cα atoms of the protein molecule and the C atoms of the drug molecule harmonically constrained (with a spring constant of 1000 KJ/mol/nm^2^). Long-range electrostatic interactions was handeled by particle-mesh Ewald electrostatics [35] with real space cut off fixed at 12 Å, and the highest magnitude of wave vectors used in reciprocal space is controlled by Fourier spacing with a parameter held at 1.2 Å.

Finally 5 ns CMD run was performed without any constraints. The CMD run was performed at 300 K and a pressure of 1 atm in the NPT ensemble with periodic boundary conditions. A time step of 2fs was used to integrate the equation of motion.

### 2. SMD Simulations

In order to perform SMD simulations, a harmonic constraint (with a spring constant κ = 1686.75 KJ/mol/nm^2^) was attached to the COM of the drug molecule and was pulled with a constant velocity, ν = 5×10^−4^ nm/ps. The chosen values are two orders of magnitude larger than those used in a typical atomic force microscopy (AFM) experiment and are akin to stiff spring approximation [36], [37]. The chosen velocity ensures that the drug molecule, during the 5 ns simulation time, traverse a distance 2.5 nm, which is more than the 2 nm AChE gorge length. Whereas, the high value of the spring constant ensures a finer spatial resolution as the thermal fluctuation of the constrained drug COM coordinate is limited to 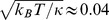 nm.

During SMD pulling, the harmonic pulling potential experienced by the drug molecule is given by.
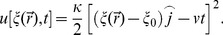
(1)


In this expression, 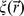 is the COM of the drug molecule relative to that of the protein molecule and 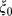 is the same quantity at t = 0. In a pulling simulation only the spring is controllable and 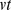 can be regarded as position of control parameter at any time t. The position of COM of the drug molecule, 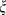 to which the spring is anchored to is however, free to choose any value depending on the nature of protein-drug interactions. Thus PMF, that we plan next to extract from SMD trajectories, can be defined both along the path of the control variable, λ ( = 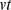) and as well as along the path of fluctuating molecular position, 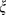. The normalized vector 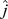 dictates the direction of pulling. We choose a direction of puling that leads from COM of active triad residues, located at the bottom of the gorge through the gorge opening (COM of the PAS residues). In order to find out if there is any other possible exit routes of the drug, we simulated the unbinding event directed both along the left and right directions of our chosen path. Within the stipulated simulation time (5 ns), we found that the drug molecule could not completely unbound through any one of these new paths. Accordingly, our chosen path is mostly the correct pathway for Ortho-7 entering and leaving the gorge.

In the pulling simulations, along with drug molecule the protein molecule may also drift away from the centre of the simulation box. One way to prohibit the drift motion is to constraint the Cα backbone of the protein molecule. This although allows specific interactions between the side chains of the amino acid residues and the drug molecule take place normally, keeping all the Cα atoms constrained has recently been shown to have a significant effect on the potential binding pockets on protein surface [38]. In view of this, in the present work, we have not applied any restraints what so ever to the backbone atoms of the protein molecule and have utilized the reference_group option of AFM pulling code of the GROMACS 3.2 suite in order to monitor the relative separation between the COM of the drug and the protein molecules. This option is set to the whole of the protein molecule with respect to which the drug molecule is being pulled. In addition, the COM motion removal facility of the Gromacs suite has also been utilized at every 500 ps interval in order to ensure that the protein molecule stayed at the centre of the simulation box during the course of the simulation.

**Figure 2:**
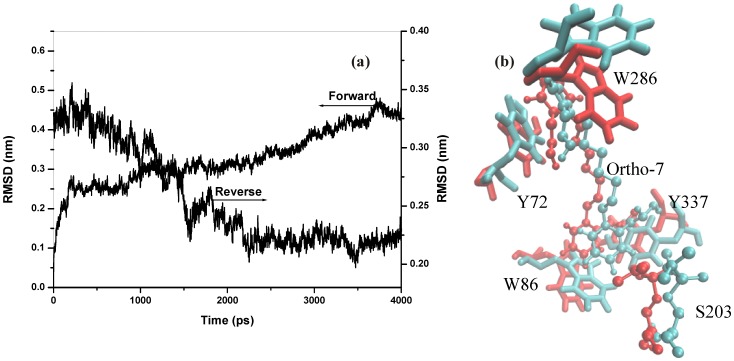
Recovery of the bound state during reverse SMD simulation. (a) All atom rms deviation of Ortho-7 and the protein binding pocket (see text later) from the crystal structure during a typical unbinding (forward) and binding (reverse) transitions of the drug molecule from the protein binding pocket. At ∼3.45 ns, the deviation attains a minimum during the reverse simulation. (b) superposition of reverse simulation snapshot (cyan) at 3.45 ns with the crystal structure (red) is shown for the drug molecule along with the key cation-π forming and S203 residues.

In order to sample the ensemble, multiple bidirectional SMD simulations of the drug molecule translocating past the same section of the gorge were performed. We carried out 10 SMD simulations, each of 5 ns duration in each of the forward and reverse directions, totaling a 100 ns run. Pulling along forward direction were initiated from choosing snapshots of CMD run separated by 0.5 ns at equilibrium. Each of the pulling along reverse direction was started from a configuration prepared by a 50 ps equilibration of the end-point structure of the corresponding forward pulling simulation. It is well known that during PMF calculations based on unidirectional SMD simulations, convergence properties of work exponential averages (see later in the formulation of reconstruction of PMF) are notorious. However, estimates of PMF from the bidirectional SMD simulations utilizes the idea that forward and reverse realization come in conjugate pairs related to time reversal, such that, what could be rare trajectory in forward pulling is a frequent trajectory in reverse pulling and vice versa. This leads to an efficient sampling and a rapid convergence and works satisfactorily in any pulling situation, from nearly equilibrium to strongly dissipative regime [22], [23]. The present work is designed along this idea and we have checked that reported PMF results converges within 8 forward/reverse realizations, while further realization up to 10 data sets only smoothened the plots. During reverse SMD simulations, the time dependence of the root-mean-square deviation (rmsd) from the X-ray crystal structure of Ortho-7 and the protein binding pocket was examined to determine whether the Ortho-7 could recover its bound state. The data is presented in Fig. 2 (a) for one of the typical binding transitions. The rmsd plot during reverse SMD simulation demonstrates that at ∼3.45 ns, Ortho-7 is the closest to its original position and orientation when compared with the X-ray structure of the bound complex. The superposition of the snapshot structure at this time with the crystal structure of Ortho-7 and a few key residues of the protein binding pocket is also shown in Figure 2 (b).

### 3. Reconstruction of the PMF

Bidirectional SMD protocol relies directly on the Crooks fluctuation theorem, which can be formally stated as: given two systems “a” and “b” described by the Hamiltonians H_a_ and H_b_, the equilibrium free energy difference, ΔF between the two states can be extracted from nonequilibrium work measurement through [26],
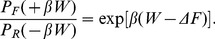
(2)


Here W is the external work done on the system forcing it to undergo 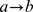 transition. *P*_F_ and *P*_R_ are the probability distribution of releasing the work W into the system during the forward (F) 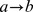 and reverse (R) 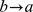 transitions, respectively in a finite time τ. For unidirectional realizations of these transitions, as monitored by the control parameter, λ, Eq. (2) can be integrated both sides to yield Jarzynski equality [25] for free energy differences between two end states. The Jarzynski relation can be further recast into the following expression for forward and reverse biasing and are given, respectively as.
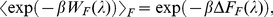
(3)


and.
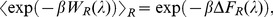
(4)where the symbol 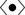 signifies path ensemble averages, 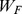
and 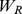 are work done on the system and 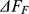 and 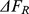 are free energy differences measured along the control parameter, λ during the forward and reverse realizations. In Eqs. (2)–(4) 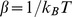. The free energies in Eqs. 3 and 4 represent HS- like estimates [27], [28] that one obtains through weighted histogram analysis method (WHAM) (see Eq. 8).

The applicability of Jarzynski relations, Eq. (3) and (4) is however seriously limited by its poor convergence properties as exponential averages hardly samples the importance region of the integral. To this effect, BAR method can be used to minimize the variance of the estimate by solving the following equation for ΔF.
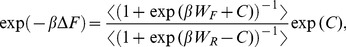
(5)where 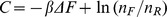, n_F_ and n_R_ are the number of forward and reverse realizations. Given that C depends on ΔF, this equation is solved iteratively and self-consistently by choosing a starting value for C, say C = 0. Clearly, the BAR method [29] is very efficient to calculate the free energy differences between the end states, “a” and “b”. However, λ can take any values between the end states, λ_a_ and λ_b_. To this effect, two different estimates of PMF as a function of λ are proposed recently. Minh and Adib [22] (MA) started from Crooks fluctuation relation (Eq. 2) and adopted (WHAM) [39] in order to connect the different thermodynamic microstates in between and arrived at an estimator,
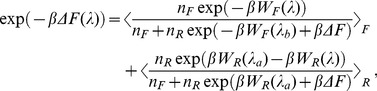
(6)where WF(λ) refers to work done to move the system from 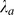 to λ in the forward direction and similarly WR(λ) is the work related to switching from λb to λ in the reverse direction. ΔF(λ) is the free energy difference between equilibrium states defined at any given intermediate time and the initial time.

In Eq. (6) ΔF, the free energy difference between the end states has to be obtained by solving Eq. (5). Note that Eq. (6) reduces to Eq. (5) at the end states. Chelli and Procacci (CP) [40] provided another estimate of PMF,
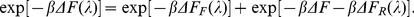
(7)


In this expression, 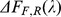
are to be evaluated following Jarzynski equality theorem (Eqs. 3, 4). The applicability of Eq. (7) spans the entire regime of pulling conditions: in the quasi reversible region it reduces to Jarzynski equality while in highly irreversible regime it behaves as MA estimator.

In a pulling simulation one often seeks an estimate of free energy of the unperturbed system as a function of collective coordinate, ξ, viz. the COM separation between the protein and the drug molecule (c.f. Eq. 1). The measurement of PMF is denoted as G(ξ). In the limit of stiff spring, since the difference between the measurements of the controlled distance and fluctuating molecular distance is negligibly small, G(ξ) and ΔF(λ) coincides. However, in the limit of soft spring, the measured difference of distance is appreciable and hence there is need of rigorous method to formulate separate expression for G(ξ). In this regard, for unidirectional biasing, HS in their pioneering work [27], [28] starting from Jarzynski equality and with the help of WHAM prescription arrived at a formula for G(ξ).
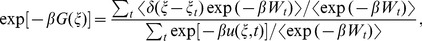
(8)where t is bin sizes over time. Equation (8) with the help of Eqns. (3) and (4) can be readily recast into PMF estimators for forward and reverse biasing, respectively.

In order to improve upon the convergence properties of HS estimator, MA [22] have included trajectories from reverse biasing into the HS estimator. The resulting PMF estimator for bidirectional simulation is given by,
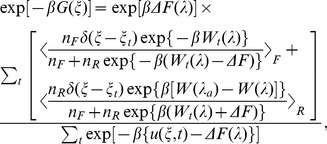
(9)where ΔF(λ) can be obtained by Eq. (6) and ΔF via BAR method, viz. Eq. (5). Note that again these set of equations have to be solved self consistently. One can also readily recast the CP formalism for the ΔF(λ) (cf. Eq. 7) into its unperturbed counterpart, G(ξ) simply by resorting to HS weighted histogram expression in place of Jarzynski equation based expression for ΔF. This results into the bidirectional PMF estimator [23],
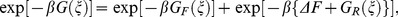
(10)where 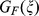 and 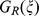 are to be evaluated from Eq. (8) for forward and reverse pulling, respectively.

The work transferred to the system and time dependent positions of the control variable and collective variable are the only inputs from simulations to these PMF estimators. In the bidirectional SMD simulations of the protein-drug complex, the results of which are discussed below, we have evaluated the work from the force-extension contour integral via Riemann sum method. Finally the initial energy of the spring, 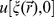 is subtracted to obtain the net work imparted on the system.

## Results and Discussion

### 1. Translocation Energetics and the Unbinding/binding Transitions

**Figure 3:**
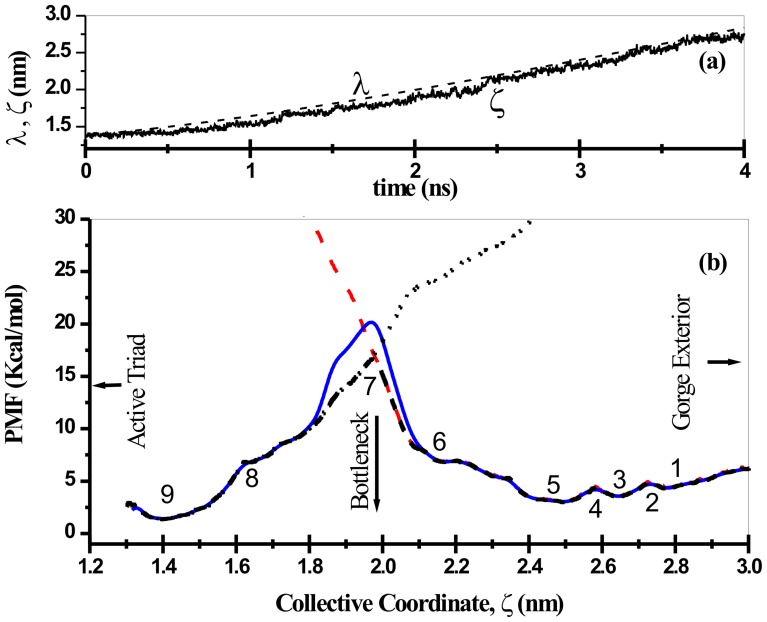
Time evolution of the instantaneous values of control parameter (λ) and the collective variable (ξ), the COM separation between the positions of the drug molecule and the enzyme is presented in panel (a) for a typical unbinding transition. The difference in sojourn of λ and ξ makes it necessary to reconstruct PMF as a function of ξ, the profiles of which are presented in panel (b); red dashed line is for binding transition, black dotted line is the profile for unbinding transition [obtained using Eq. 8]. The dashed black line and solid blue lines are obtained from bidirectional PMF estimators, Eq. 10 and Eq. 9, respectively.

PMF as a function of real molecular collective coordinate was successfully reconstructed from bidirectional SMD simulations, (see Fig. 3). For the sake of comparison, PMFs as obtained from unidirectional estimators (cf. Eq. 8) for forward/reverse methods are also presented. Clearly the unidirectional methods overestimate the PMF in the long run as the system is progressively driven away from its equilibrium configuration. Bidirectional estimator, on the other hand, that utilizes the trajectories in the two directions is in effect reduces the nonequilibrium bias and therefore can construct the true PMF of the protein-drug system investigated. The accumulated work (data not shown) which were evaluated from force profile are on an average growing function of time (and hence positions of drug molecule) in a constant velocity SMD simulation. The bidirectional PMF estimators effectively recombines forward-reverse trajectories to arrive at a converged work-position relationship and hence could arrive at a fuller estimate of equilibrium free energy from the measured nonequilibrium works, free from nonequilibrium bias, if any, from either directions. As one would naively expects, except in the case of the infinitely stiff spring, the control variable may or may not follow the path traversed by the molecular collective coordinate of our interest during the biased transitions. One such typical mismatch between their values are plotted in Fig. 3(a) for unbinding transition of the drug molecule. Such a mismatch necessitates the formulation of PMF as a function of ξ and the results are presented in Fig. 3(b). The energy basins and barriers (marked as 1 to 9) to the drug unbinding/binding transitions are also shown.

**Figure 4:**
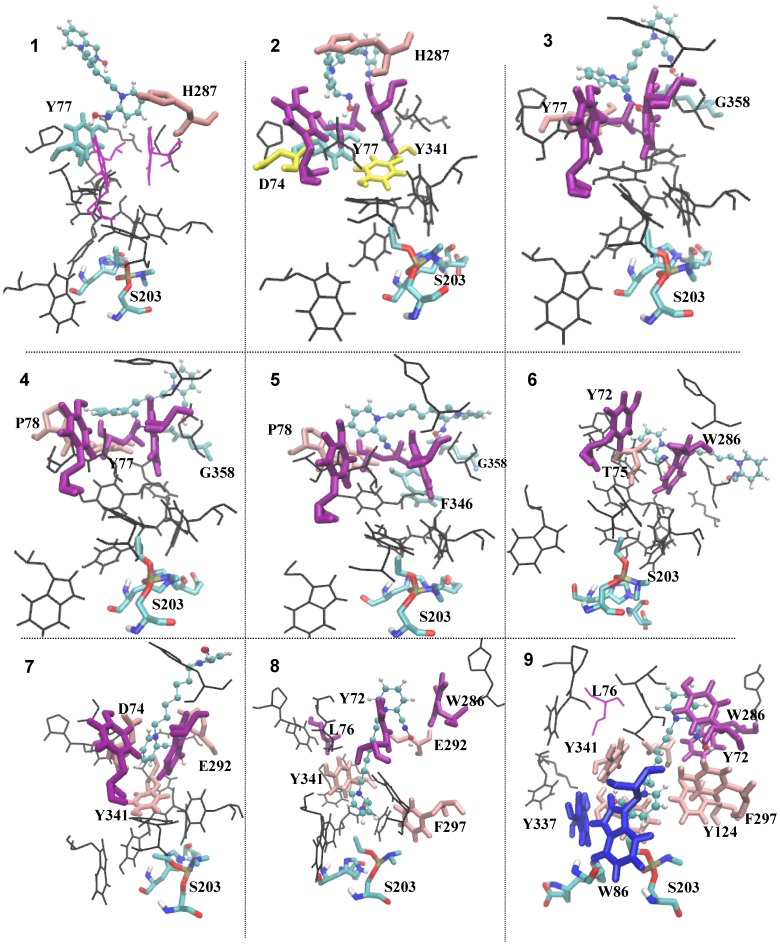
Snapshots of representative SMD trajectories for the basins and barriers 1–9 as marked in Fig. 3 are shown perpendicular to the pulling direction. Only those residues of AChE which forms the binding pocket of the enzyme and are responsible for the drug capture and release are shown. Residues responsible for hydrophobic interactions are shown in pink, whereas residues forming hydrogen bonds with the drug are colored in cyan. In purple are PAS residues Y72, W286 that along with L76 forms the bottleneck of gorge which the drug molecule has to push apart in order to gain access to active triad (S203 shown in the bottom of the gorge). Residues Y72 and W286 forms strong cation-π interaction with the pyridinium rings of the drug in two stages: first with the active pyridinium ring, when the drug is first captured from the environment (snapshot 6) and second with the peripheral pyridinium ring when the drug is fully bound to the gorge (snapshot 9). In the fully captured state, residues W86 and Y337 (shown in blue) also forms similar cation-π interaction with the active pyridinium ring. D74 and Y341 (shown in yellow) forms a key hydrogen bond forming/breaking pair due to which the drug molecule gain access to the bottle neck region.

The highest barrier (point 7) in the PMF profile corresponds to the drug COM position (relative to the protein COM) at which the COM of bottleneck of the protein (comprising of residues L76, Y72 and W286) also resides [41]. Interestingly the unidirectional PMF estimates for unbinding and binding transitions cut across each other at this point. This feature of PMF estimates obtained through unidirectional method also help identifying the binding and unbinding transitions in the bidirectional PMF profile. For example, the PMF profile as obtained from binding transitions (reverse SMD) of the drug molecule coincides well with the bidirectional estimates of PMF and therefore the basin (points 1, 3, 5, and 6 in Fig. 3b and 4) and barriers (points 2, 4, and 7 in Fig. 3b and 4) to the right hand side of the bottleneck position can be ascribed to those due to binding transitions leading to the bottleneck of the gorge. Similar to this, the points 9, 8, and 7 (in Fig. 3b and 4) can be referred to those transition events leading to unbinding of the drug molecule. In view of this, the free energy barrier to the unbinding of the drug molecule from the active-site to the bottleneck of the tabun conjugated mAChE is estimated to be ∼13.3 kcal/mol, whereas that due to binding event is ∼9.5 kcal/mol. An inspection of the force profile also reveals the force involved during unbinding transition is larger than the force required to bind the drug molecule. This difference in forces which eventually are reflected in the measured PMF clearly suggests the distinction between the process of Ortho-7 binding and unbinding. Bidirectional PMF estimators could also identify the barrier offered by the bottleneck region towards the drug binding/unbinding; as the drug molecule that is captured from the gorge exterior could pass through the bottleneck will experience a downhill potential and eventually be tightly held in the active-site region, which also corroborates well with the crystallographically resolved equilibrium structure [10]. The difference in the PMF values (see Fig. 3b) obtained through Eq. 9 and Eq. 10 is due to peculiarities related to the evaluation of work exponential averages that has been dealt with in detail in ref. 23. In particular, it is to be noted that Eq. 10 is superior than Eq. 9 in two extreme cases: one in which the pulling conditions are in the highly dissipative regime and the other when the number of realizations employed in the path-ensemble averages of the estimator is small.

**Figure 5:**
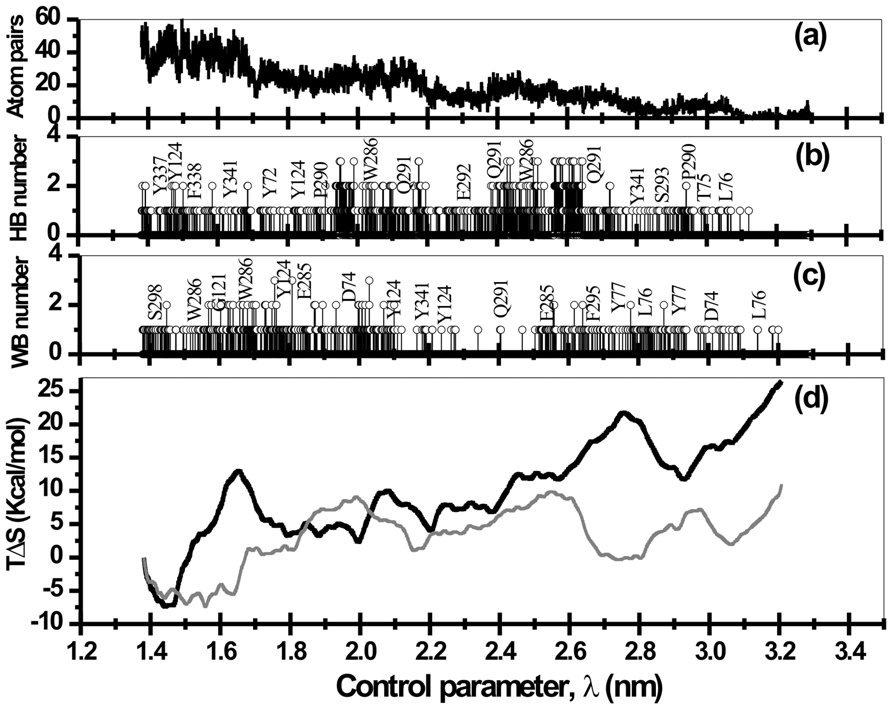
Number of hydrophobic atom pairs (a), protein-drug direct hydrogen bond numbers (b), number of water bridges that interconnects the protein with the drug molecule through hydrogen bonds (c), and the entropic control of unbinding transitions at 300 K (d) are presented as a function of control parameter, λ. Entropy estimates of the unbinding transition in which the solvent translational mobility is inhibited is presented in gray line in panel (d).

**Figure 6:**
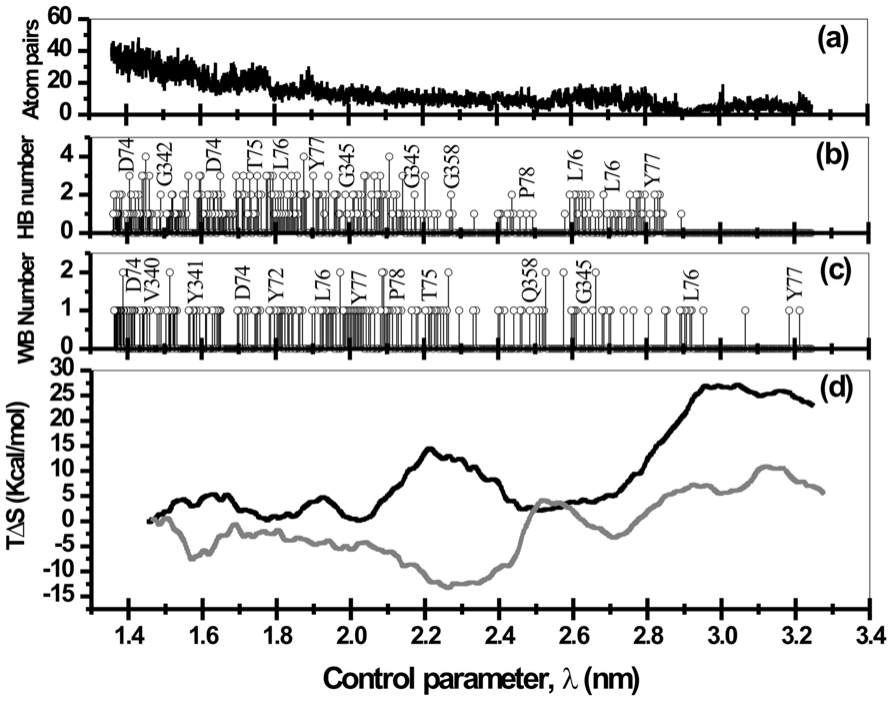
During the binding transitions the number of hydrophobic atom pairs (a), protein-drug direct hydrogen bond numbers (b), number of water bridges that interconnects the protein with the drug molecule through hydrogen bonds (c), and the entropic control at 300 K (d) are presented as a function of control parameter, λ. Entropy estimates of the binding transition in which the solvent translational mobility is inhibited is presented in gray line in panel (d).

The sojourn of the drug molecule from the gorge exterior or from active-site region into the bottleneck region is accompanied by several well orchestrated events involving several structural and functional features of the protein-drug interactions. Snapshots of SMD runs corresponding to the minima and maxima in Fig. 3 are depicted in Fig. 4. The main protein-drug interactions considered here are DHB, WB and HI. As the drug molecule slides in/out of the gorge, it encounters several favorable interactions of these sorts that capture the molecule in local minima characterized by long residence times. During the course of the sliding motion, older interactions break up in search of newer ones while passing through the barriers. This space (time) dependent interaction profiles are presented in panel (a), (b), and (c) of Figs. 5 and 6 for typical unbinding and binding transitions, respectively.

The occurrence of DHB, WB, and HI are often complementary to each other. For example, when DHB between Ortho-7 and mAChE begins to break, the energy loss due to it is being compensated partially by increased hydrophobic interactions and formation of new WB. The final position of Ortho-7 (point 9 in Fig. 3b and Fig. 4) is stabilized by a host of suitable hydrophobic interactions with aromatic residues lining the gorge wall, DHB and WB interactions in addition to the strong cation-π interactions [10] with Y72, W286 and W86, Y337 pair of aromatic residues. An analysis of the unbinding trajectories further reveals that untrapping of the drug molecule from this final position involves breaking of the cation-π interaction of active-pyridinium ring of Ortho-7 with the W86 and Y337 residues (point 8 in Fig. 3b and Fig 4) while the old cation-π interaction between the peripheral pyridinium ring and PAS residues Y72 and W286 still prevails. The increase in free energy in this process is compensated by hydrophobic interactions with E292, Y341, and F297 along with WB interaction with W286 and Y124 (see Fig. 5). As the unbinding transition continues, the highest free energy barrier is encountered when the COM separation between the drug and the protein is ∼2 nm (point 7 in Fig. 3b and 4) and corresponds to the bottleneck region of the gorge. At this point, the active-pyridinium ring of Ortho-7 lies in plane with the aromatic rings of the bottleneck residues Y72 and W286 forming only one cation-π interaction in place of two (compare with point 9), while the rest of the drug molecule is almost out of the binding pocket. The energetic cost associated with the breaking of the old HI (corresponding to point 8 in Fig. 4) is compensated by newer one involving Y341, E292 and D74 residues.

When viewed in terms of binding transitions, the major effort to the Ortho-7 entry into the gorge (point 6 in Fig. 3b and 4) is to cross the plane composed by the bottleneck residues. The passage of the drug molecule from the free energy minima (point 6) in which it lies on the surface of the bottleneck region to an orientation benign for further penetration into the gorge (point 7) is energetically driven by favorable cation- π interaction and DHBs (pertaining to the highest free energy barrier) as mentioned above. It is to be noted that unlike metadynamics simulations in which the cation-π interactions are directly measured [20], we inferred our result based on measured minimal Van der Waals separation between the pyridinium moieties of the drug molecule and the aromatic rings of the cation- π interaction forming residues (data not shown), since the cation-π interaction parameter is a function of intermolecular separation vector.

A favorable orientation of the active pyridinium ring of the drug molecule such that it can form HI interactions with H287 and the oxime oxygen of the drug molecule can participate in DHB with Y77 helps the gorge to snatch the drug molecule from environment while it is nearby (point 1 in Fig 3b and 4). The second energy basin (point 3) occurs at about 2.65 nm caused by the favorable DHB with G358 and hydrophobic interaction with Y77. The passage of the drug molecule from basin 1 to basin 3 through the intervening barrier (point 2 in Fig. 3 and 4) is associated with breaking of a key inter-residue hydrogen bond between D74 and Y341. Similar such key inter-residue hydrogen bonding pair, D74-Y341 was also noticed responsible for tetramethyl ammonium ligand entry into mAChE gorge [20], [21]. As this hydrogen bond breaks, the active pyridinium ring of Ortho-7 gains access to the bottleneck region and the cation-π interaction with W286 starts forming (point 3). Further access to the gorge (points 4, 5, 6 in Fig. 3b and 4) involves suitable orientation of the active pyridinium moiety with respect to the plane of the cation-π interaction forming residues W286 and Y72 while the drug molecule is still in the bottleneck region forming DHB with G358, F346 and hydrophobic interaction with P78 and T75.

From repeated SMD runs we found that passage of the drug molecule in this region (from point 4 at ∼2.6 nm to point 6 at ∼2.2 nm) the orientational flexibility of the drug molecule remains quite high. However, at the major barrier (point 7 in Fig. 3b and 4) it loses its orientational flexibility when the active pyridinium moiety forms strong cation-π interaction with the bottleneck residues, W286 and Y72. An estimation of entropy as a function of control parameter, λ (see later in the text and Fig. 6d) also reveals that the drug molecule continuously losses its configurational entropy during its binding transition corresponding to the point 1 to point 4 and then increases again for its journey from point 4 to point 6 reflecting the aforementioned orientational flexibility. Thereafter as the binding transition continues, the configurational entropy attains a minimum at ∼2 nm corresponding to tight holding of active pyridinium ring by the W286 and Y72 cation-π interaction forming residues. As a result of this entropic penalty the major barrier also occurs at ∼2 nm. A similar estimate of entropies of unbinding transition (see Fig. 5d) also reveals that immediately after release of drug from basin 9, its entropy increases suggesting some degree of flexibility of the drug molecule in the enzyme binding pocket. However, for the control parameter corresponding to the journey from point 8 to point 7 (see Fig. 3b) the entropy of unbinding transition decreases reaching a positive minimum at the major barrier.

### 2. Entropic Control

During translocation through the binding gorge of the enzyme, the drug molecule undergoes dynamic interactions, such as DHB and HI, as detailed above. The water molecules that interconnect the drug and the protein through shared hydrogen bonds (i.e., water bridges) are also translationally mobile. As a result, like DHB and HI, water bridge interactions are also dynamic in nature. All these interactions are likely to result into loss of configurational entropy of the drug-protein binding pocket entity and hence causes the entropic penalty towards their binding free energy.

In order to decipher the residues responsible for such interactions, we have analyzed every possible atom pairs forming DHB, HI, and WB as a function of the control parameter, λ. From an analysis of these interactions (c.f. Figs. 5 and 6 where only the key residues are mentioned), the binding pocket of the enzyme is found to be composed of residues 72, 74–78, 86, 121, 122, 124, 125, 203, 204, 286, 287, 290–295, 297, 298, 334, 337, 338, 340–346, 358, 361, 365, and 447. These residues along with the drug molecule are comprised of a total 456 atoms that we define to form a drug-protein binding entity.

The time dependent SMD trajectories of the binding entity from where these protein-drug interactions are extracted can also be utilized to arrive at an estimate of entropic contribution, S(λ) to the binding free energy [42] as a function of control parameter, λ. We adopt the quasiharmonic (QH) formulation [43] to estimate S(λ). QH method involves carrying out MD simulation, computing the covariance matrix of atomic coordinates, interpreting the covariances, computing effective or quasiharmonic force constants to finally compute approximate values of entropy. The computed entropies are approximate [44] because QH method inherently merges multiple free energy wells into a wide energy well whose entropy is obviously much greater than the actuality. Also, while constructing covariance matrix elements,
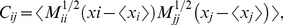
(11)


the use of atomic positions in Cartesian basis set [45] can lead to serious error in the computed classical entropies [46] evaluated through the relation,
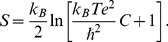
(12)


This is because a small change of an internal coordinates necessarily involve multiple Cartesian degrees of freedom and hence would lead to higher entropy estimates. In Eq. 11, M is a diagonal mass matrix of the constituent atoms. The use of Cartesian basis set is however, allowed and the error due to this can be minimized provided one uses the formula for quantum mechanical entropy [45],
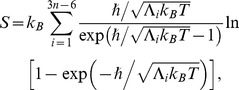
(13)where 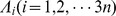 are the eigen values of covariance matrix C. The genesis of either of the entropy formulas, Eq. (12) or (13) can be traced back to the relation,
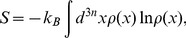
(14)where 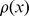 measures the fluctuations observed in the coordinate space during an equilibrium MD simulation. For a nonequilibrium SMD simulation, one can imagine to formulate a similar expression [42] for entropy as a function of time.



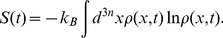
(15)In order to further improve the accuracy of the estimates [42] one can further recast Eq. (15) into.
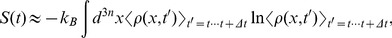
(16)where 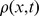 is now measured for the subensemble of trajectories between time t and t+Δt, whose covariance and hence their eigen values will also be time dependent.

Given this time dependent formulation, Eq. (13) can be rewritten for the relative entropy estimate with respect to the bound state of the drug to be given by.
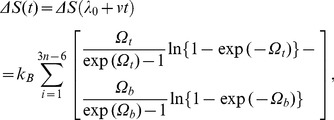
(17)where 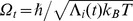 and 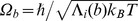. In Eq. (17) while eigen values 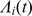 are to be evaluated for the subensemble of trajectories between t and t+Δt, the eigen values 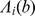 are to be evaluated from trajectories of the bound state of the drug molecule. We will use Eq. (17) to calculate the entropic contribution towards free energy profile during drug binding/unbinding transitions.

However, it is to be noted that the nonequilibrium SMD trajectories to be used here to extract entropies suffers from sampling error because the phase space region visited within the short simulation time may be inadequate. To minimize this error [42] we use ten SMD trajectories for each of the bidirectional simulation pathways. From each of the 5 ns simulation, 5000 coordinate sets of the binding entity were taken for covariance analysis. Next we divide 5000 coordinate sets, each of which were of 1 ps duration, into 230 overlapping subsets having a temporal width of 20 ps, while each subsets were of 400 ps duration. For the SMD velocity of the control parameter equal to 5×10^−4^ nm/ps, the 20ps overlapped region between the 400ps subintervals refer to 0.01 nm and 0.2 nm shift of the control parameter, respectively. This ensures that the length of the phase space covered during 5 ns simulation is 230×0.01 nm+0.2 nm = 2.5 nm, which is larger than the length of the gorge. The 230 subsets of 400 trajectories were then subjected to covariance analysis and entropies were evaluated following Eq. (17).

The estimated entropies are presented in Fig. 5 and 6 for forward and reverse bias, respectively. Clearly as the drug molecule escapes from the binding pocket, the entropy increases (Fig. 5) while during binding the restriction of its motion within the binding pocket causes a loss of configurational entropy (Fig. 6). The fluctuations in the measured entropy are mere reflection of several stages of binding/unbinding steps (c.f. Fig. 4) during the drug translocation. The highest free energy barrier to the unbinding pathway is ∼8 kcal/mol which occurs at λ ∼1.85 nm (data not shown). The positive entropic contribution towards this binding event is ∼5 kcal/mol. The initial rapid increase of entropy up to λ∼1.65 nm during unbinding transition (see Fig. 5) can be associated with the release of peripheral and active pyridinium rings of the drug molecule from cation-π interacting aromatic residues at the PAS and at the active-site region, respectively. Immediately thereafter the entropy values drops indicating that the active pyridinium ring of the drug is now sandwiched by the PAS residues forming cation-π interaction with Y72 and W286 causing a loss in configurational entropy. At the unbinding free energy barrier at ∼1.85nm, both the pyridinium rings are out of the PAS and entropy values steadily increases with intermediate fall and rise caused by a variety of interactions with the residues lying in the surface of the bottleneck region (c.f. Fig. 4).

It can be seen in Fig. 6, as the drug molecule binds to the active-site gorge the configurational entropy of the binding entity steadily decreases and free energy barrier towards binding increases. The computed loss of configurational entropy up to λ∼1.85nm is large (∼18 kcal/mol) compared to the binding free energy of ∼3 kcal/mol after the drug molecule is captured from the environment. This huge loss of entropy is however not unusual [42], [44] and can result from narrower energy wells leading to the bound state. In addition, the usual caveats against extracting configurational entropy from SMD simulations, as detailed in ref. 42, also apply in our present formalism.

**Figure 7:**
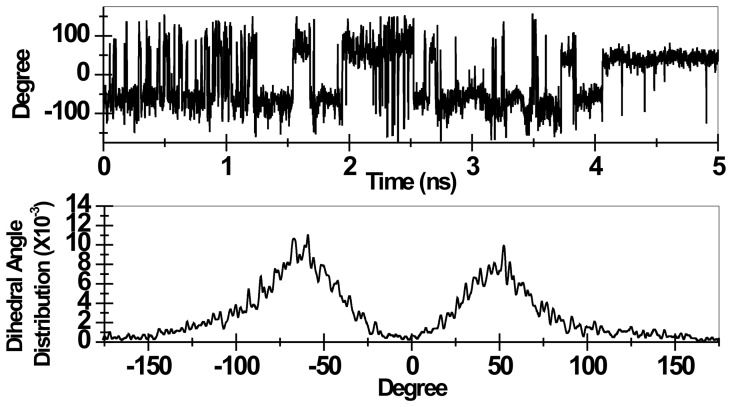
Temporal fluctuation of the torsion between oxime and pyridinium ring (dihedral: N9-C8A-C7-N2) is presented during a typical binding transition of the Ortho-7 (upper panel). Dihedral angle distribution of the said fluctuation is presented in lower panel.

In contrast to second-generation mining minima algorithm [44], the QH model cannot decompose the obtained configurational entropy into dihedral, angular and stretching components. However, one particular torsion, namely the torsion between the oxime and the pyridinium ring (termed as torsion O) plays a vital role for the effective reactivation of the tabun conjugated serine [10]. Therefore it is natural to seek for an estimation of this dihedral angle variation with respect to time. In Fig. 7 the temporal profile of torsion O along with the torsion angle distribution is presented. Clearly torsion O undergoes rapid fluctuations and its contribution towards configurational entropy would be significant. The dihedral angle distribution, extracted from temporal profile, is found to be peaked at around ±60 arc degrees. A similar analysis on the dihedral angle distribution between 2-substituted oxime and the pyridinium ring of another poorer oxime drug, HLÖ-7 is found to be peaked at around ±90 arc degrees (data not shown). Both these observations corroborate well with experimental findings and might explain the relatively higher (albeit poor) efficacy of Ortho-7 among the two oxime drugs, since the lower value of the torsion O in the case of Ortho-7 would also keep the potential energy of the reactivation transition point relatively low [10].

### 3. Role of Water in Drug Translocation

The role of water molecules in the protein dynamical transitions and hence the capture of drug molecule from environment and its subsequent translocation to the active-site of the protein has remained an active research for many years. These issues has recently been probed through MD simulations [47], [18]. It has been argued [18] that the buried water molecules in the enzyme gorge acts as a “lubricant” that facilitates the passage of the drug molecule to the active-site. Clearly water molecules on the surface of the protein and those buried inside the gorge participate in the HB making/breaking process with protein residues and also with the drug molecule. In the absence of water molecule, such as in a dehydrated system, the protein molecule loses its flexibility due to strong electrostatic intra-residue and protein-drug inter-residue interactions. The intervention of water molecules breaks up these interactions, protein loses its rigidity, and eventually orchestrate the passage of the drug molecule through the formation of WB and useful protein-drug HB (cf. Figs. 5 and 6). It has been demonstrated in ref. 47 that effects of artificially inhibiting the solvent translational mobility is equivalent to a dehydrated system. Inspired by this work and our expectation that water molecules acts as “lubricant”, we have explored the role of water molecule in computed entropy and PMF of binding/unbinding transitions through bidirectional simulations in which the water molecules were partially immobilized.

**Figure 8:**
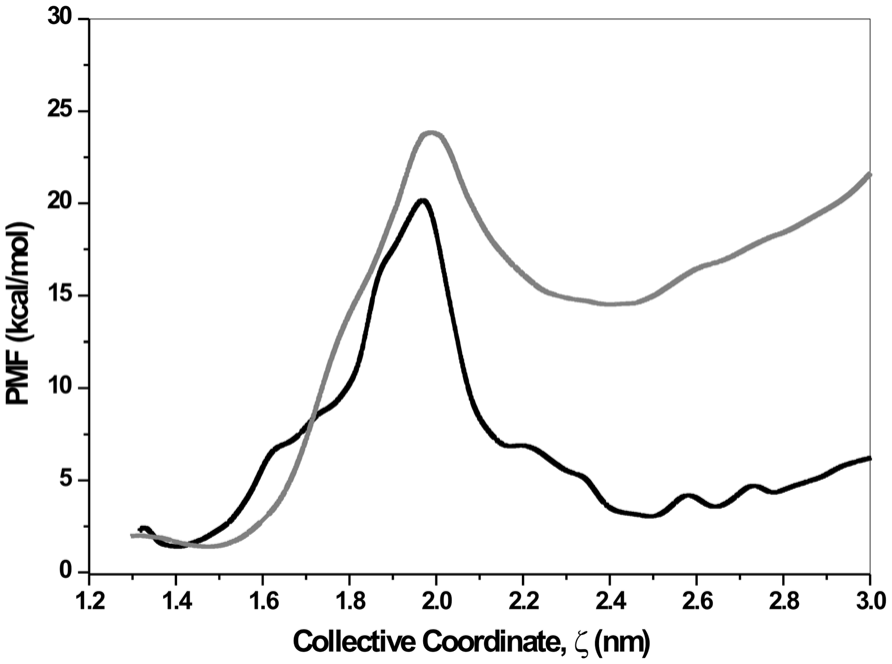
The PMF calculated using Eq. 9 and bidirectional SMD simulations of the mAChE-tabun.Ortho-7 complex dissolved in water is compared for two cases: one in which the positions of water O atoms are restrained by a harmonic potential (gray line) and the other where there is no such restraints (black line, same as Fig. 3b).

We performed the same set of bidirectional simulations of the complex dissolved in water with one exception; O atoms of water molecules are now restrained by a harmonic potential with a force constant equal to 60 KJ/mol/nm^2^. Use of this force constant [47] ensures that the water molecules preserve its rotational freedom while it loses its translational mobility by factor of ∼2 (when compared with an unrestrained situation as we dealt with above). Since rotational freedom is kept unaltered so also are the average protein-water HB lifetime are least affected. However, partially immobile water molecules make the protein-water HB, and hence, protein-drug direct HB and WB network relaxation time longer. As a result, the entropic penalty towards drug binding and unbinding will be even more severe compared to an unrestrained situation. Entropy estimates presented in panel (d) in Figs. 5 and 6 for solvent restrained case vindicate this expectation. As can be seen in these figures the entropy estimates can even become negative. Further, force exerted on the system during SMD simulations also increases (data not shown). This resulted into a higher PMF as presented and compared in Fig. 8.

It is interesting to note that the main barrier to the drug binding remained unaltered at the bottleneck region. However, free energy barrier to drug uptake from the environment increases ∼8 times as compared to the unrestrained situation, To find the root cause behind this we analyzed the intra-residue HB and protein-drug WB during the initial phases of drug uptake. It was found that the temporal features of key interactions responsible for initial uptake of the drug molecule like the HB and WB between drug and Y77, L76 residues and a key protein inter-residue HB between D74 and Y341 changes markedly. Namely, the time interval between breakage and reformation of HB and WB with the same water molecule is narrowed down significantly resulting into rigidified protein-drug interactions. Whereas the protein-drug HB and the HB between D74 and Y341 survives up to 1ns with rare intermittent breaks. Thus the effect of partially immobile water molecule can be interpreted as enhanced protein-drug interactions at the cost of a normal relaxation of these interactions that occurs in an unrestrained situation (see Figs. 5 and 6), which subsequently allow easier uptake of the drug molecule from environment.

In order to investigate further on the possible role of solvent mobility on the structure of the translocation channel, we have also calculated the radius of gyration (ROG) of the protein binding pocket during binding/unbinding transitions of the drug molecule for both the restrained and unrestrained situations. During unbinding transitions (data not shown) we found that the ROG value (a measure of the compactness of structure) that starts from ∼1.8 nm expands up to 1.98 nm in an unrestrained water environment, while that in a restrained environment the ROG values fluctuates in and around 1.8 nm. Similar results holds good for binding transitions also. The compact gorge structure may also be interpreted to give rise to a higher free energy barrier during drug translocation in a restrained water environment.

### Conclusion

Using SMD protocol we have conducted complete and continuous translocation of one of the most potent oxime drug, Ortho-7 through the active-site gorge of tabun intoxicated mAChE. Based on bidirectional estimators that effectively combine position and work measurements obtained during nonequilibrium unbinding-binding transitions, we have reconstructed the protein-drug PMF along the translocation pathway. The obtained PMF as function of molecular collective coordinate (or the control parameter) is superior than those obtained through unidirectional estimators and the binding sites and barriers along the conduction pathway could be located. An in depth analysis of protein-drug HB, HI and WB elucidates the temporal and structural features of the conduction mechanism along the gorge. The obtained PMF with respect to the bottleneck residues exhibits asymmetry towards unbinding/binding transitions. Namely, the energetic cost involved in unbinding the drug molecule is larger than capturing the molecule from environment and finally binding it to the active-site. This might explain the higher efficacy of Ortho-7 in reactivating the enzyme when compared with another less effective oxime drug, HLO-7 [10], where we found (data not shown) that the asymmetric nature of the PMF profile is reversed (as with respect to Ortho-7), namely, the energetic cost involved in binding the drug molecule is found to be larger than unbinding it from the protein gorge. Bidirectional SMD simulations and PMF reconstruction thereof seems to be an effective tool that might help designing improved nerve-agent antidotes. It is to be noted that the evaluated free energy barrier can be further utilized to draw important conclusions on the kinetics of the process provided the discovered PMF barrier can be proved to be a true transition state through an analysis of the distribution of committor at that point [48].

The same numbers of bidirectional repeated SMD trajectories used to construct PMF are also utilized to estimate the entropic contribution to the free energy along the translocation pathway. We have used the quantum mechanical entropy formula such that the configurational entropy could be extracted from Cartesian coordinate covariance analysis without requiring their conversion into internal coordinates. The loss of Ortho-7′s configurational entropy along the binding pathway is remarkably large and may therefore compensate for the enthalpic contribution to binding free energy barrier. The obtained entropic estimate is quite reminiscent of high affinity protein-drug systems that undergo similar large loss of entropy upon binding. The present result may guide future endeavor of novel antidote design where the drug molecules may be made more rigid requiring less entropy to lose upon binding. An intrinsically rigid antidote is thus expected to bind efficiently. The problem of choosing such antidotes with less degrees of freedom is certainly that they must possess the correct conformation to be able to readily bind at PAS and as well as to the active-site of the enzyme. However, caution should be exercised while considering the balance between entropic and enthalpic contributions to the drug binding, as it has been recently demonstrated that making a ligand more rigid did not necessarily increase its binding free enrgy [49].

To further investigate the role of water molecules on the energetics of drug translocation, we have conducted separate bidirectional SMD simulations in which the solvent translational mobility was inhibited. The inhibited system is equivalent to “dehydrated” protein and found to pose a rigid structure which can hardly be able to capture the drug molecule and translocate it through the gorge. This observation reaffirms the fact that translationaly mobile water acts as “lubricant” for drug capture by the surface of the bottleneck region of enzyme and subsequent translocation to the active-site.

## Supporting Information

File S1 **The topology and bonded parameters for the tabun conjugated serine (SUN) are listed.** The topology file includes the partial charges of SUN.
(DOC)
