# Supplementary material for: Energetics of Ortho-7 (Oxime Drug) Translocation through the Active-Site Gorge of Tabun Conjugated Acetylcholinesterase
Source: PLoS One. 2012 Jul 11;7(7):e40188. doi: 10.1371/journal.pone.0040188 (PMC3394793; doi:10.1371/journal.pone.0040188)
Supplement: File S1 — The topology and bonded parameters for the tabun conjugated serine (SUN) are listed. The topology file includes the partial charges of SUN. (DOC) [file pone.0040188.s001.doc]

**File S1**

Energetics of Ortho-7 (oxime drug) translocation through the active-site gorge of tabun conjugated acetylcholinesterase

Vivek Sinha, Bishwajit Ganguly, and Tusar Bandyopadhyay

**The toplogy and parameters for SUN (tabun conjugated serine) residue transcribed to GROMOS force field format**

--------


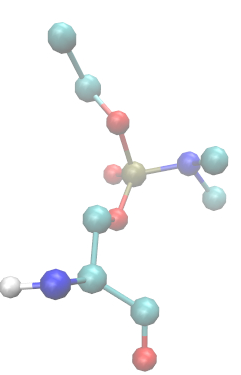


**C4**

**C3**

**O2P**

**O1P**

**P**

**N1**

**C2**

**OG**

**CB**

**N**

**H**

**CA**

**C**

**O**

**SUN Residue**

**C1**

[ SUN ]

[ atoms ]

| N | N | -0.764775 | 0 |
| --- | --- | --- | --- |
| H | H | 0.464155 | 0 |
| CA | CH1 | 0.159176 | 1 |
| CB | CH2 | 0.415592 | 1 |
| OG | OA | -0.723256 | 1 |
| P | P | 1.697372 | 2 |
| O1P | OP | -0.790076 | 2 |
| O2P | OP | -0.729938 | 2 |
| N1 | NL | -0.730563 | 2 |
| C1 | CH3 | 0.253891 | 3 |
| C2 | CH3 | 0.249578 | 3 |
| C3 | CH2 | 0.368884 | 4 |
| C4 | CH3 | 0.025629 | 4 |
| C | C | 0.753374 | 5 |
| O | O | -0.649043 | 5 |

[ bonds ]

| N | H | gb_49 |
| --- | --- | --- |
| N | CA | gb_50 |
| CA | C | gb_51 |
| C | O | gb_52 |
| C | +N | gb_53 |
| CA | CB | gb_54 |
| CB | OG | gb_55 |
| P | OG | gb_56 |
| P | N1 | gb_57 |
| P | O1P | gb_58 |
| P | O2P | gb_59 |
| N1 | C1 | gb_60 |
| N1 | C2 | gb_61 |
| C3 | O2P | gb_62 |
| C3 | C4 | gb_63 |

[ angles ]

| ; ai | aj | ak | gromos type |
| --- | --- | --- | --- |
| -C | N | H | ga_50 |
| H | N | CA | ga_51 |
| -C | N | CA | ga_52 |
| N | CA | C | ga_53 |
| CA | C | +N | ga_54 |
| CA | C | O | ga_55 |
| O | C | +N | ga_56 |
| N | CA | CB | ga_57 |
| C | CA | CB | ga_58 |
| CA | CB | OG | ga_59 |
| CB | OG | P | ga_60 |
| OG | P | N1 | ga_61 |
| OG | P | O1P | ga_62 |
| OG | P | O2P | ga_63 |
| N1 | P | O1P | ga_64 |
| N1 | P | O2P | ga_65 |
| O1P | P | O2P | ga_66 |
| P | N1 | C1 | ga_67 |
| P | N1 | C2 | ga_68 |
| C1 | N1 | C2 | ga_69 |
| P | O2P | C3 | ga_70 |
| O2P | C3 | C4 | ga_71 |

[ impropers ]

| ; ai | aj | ak | al | gromos type |
| --- | --- | --- | --- | --- |
| N | -C | CA | H | gi_1 |
| C | CA | +N | O | gi_1 |
| CA | N | C | CB | gi_2 |
| P | OG | N1 | O1P | gi_2 |
| N1 | P | C2 | C1 | gi_2 |

[ dihedrals ]

| ai | aj | ak | al | gromos type |
| --- | --- | --- | --- | --- |
| -CA | -C | N | CA | gd_22 |
| -C | N | CA | C | gd_23 |
| N | CA | C | +N | gd_24 |
| N | CA | CB | OG | gd_25 |
| CB | CA | C | O | gd_26 |
| C | CA | N | H | gd_27 |
| OG | CB | CA | C | gd_28 |
| CA | CB | OG | P | gd_29 |
| CB | OG | P | O2P | gd_30 |
| C2 | N1 | P | OG | gd_31 |
| C3 | O2P | P | OG | gd_32 |
| C4 | C3 | O2P | P | gd_33 |

; GROMOS bond-stretching parameters

| #define gb_49 | 0.10000 | 1.86912e+07 |
| --- | --- | --- |
| #define gb_50 | 0.14532 | 6.87100e+06 |
| #define gb_51 | 0.15356 | 5.16392e+06 |
| #define gb_52 | 0.15228 | 5.34761e+06 |
| #define gb_53 | 0.12054 | 1.68591e+07 |
| #define gb_54 | 0.13300 | 1.17923e+07 |
| #define gb_55 | 0.14267 | 8.06129e+06 |
| #define gb_56 | 0.15824 | 4.79120e+06 |
| #define gb_57 | 0.15783 | 5.04829e+06 |
| #define gb_58 | 0.16414 | 3.01539e+06 |
| #define gb_59 | 0.14635 | 6.75831e+06 |
| #define gb_60 | 0.14570 | 6.84373e+06 |
| #define gb_61 | 0.14560 | 6.87003e+06 |
| #define gb_62 | 0.14321 | 6.99748e+06 |
| #define gb_63 | 0.15162 | 5.27403e+06 |

; GROMOS bond-angle bending parameters

| #define ga_50 | 118.0574 | 435.92372 |
| --- | --- | --- |
| #define ga_51 | 117.3209 | 474.63920 |
| #define ga_52 | 122.2190 | 692.46402 |
| #define ga_53 | 111.7928 | 518.00520 |
| #define ga_54 | 110.2111 | 479.43792 |
| #define ga_55 | 108.6776 | 433.54712 |
| #define ga_56 | 109.7734 | 454.43691 |
| #define ga_57 | 111.0586 | 510.62302 |
| #define ga_58 | 115.2385 | 614.37121 |
| #define ga_59 | 121.0517 | 646.72301 |
| #define ga_60 | 123.6995 | 729.87329 |
| #define ga_61 | 121.6604 | 537.72301 |
| #define ga_62 | 123.1651 | 719.53621 |
| #define ga_63 | 105.2510 | 419.59128 |
| #define ga_64 | 106.6812 | 434.91534 |
| #define ga_65 | 114.0347 | 630.82300 |
| #define ga_66 | 101.4660 | 409.28461 |
| #define ga_67 | 113.9276 | 580.74012 |
| #define ga_68 | 119.6633 | 632.40214 |
| #define ga_69 | 114.4011 | 765.38730 |
| #define ga_70 | 114.3029 | 763.63801 |
| #define ga_71 | 119.0787 | 494.56212 |

; GROMOS improper (harmonic) dihedral angle parameters

| #define gi_1 | 0.0 | 167.42309 |
| --- | --- | --- |
| #define gi_2 | 35.26439 | 334.84617 |

; GROMOS (trigonometric) dihedral torsional angle parameters

| #define gd_22 | -172.0294 | 32.74125 | 2 |
| --- | --- | --- | --- |
| #define gd_23 | -86.8253 | 1.08752 | 6 |
| #define gd_24 | 69.8222 | 1.01468 | 6 |
| #define gd_25 | 10.5582 | 1.63511 | 6 |
| #define gd_26 | -60.7155 | 4.92831 | 6 |
| #define gd_27 | 176.4610 | 4.74251 | 6 |
| #define gd_28 | 111.1237 | 3.95313 | 3 |
| #define gd_29 | 109.2209 | 1.67542 | 3 |
| #define gd_30 | -95.3666 | 1.94727 | 3 |
| #define gd_31 | 69.8429 | 3.62911 | 2 |
| #define gd_32 | -54.0545 | 2.94721 | 2 |
| #define gd_33 | -156.8014 | 2.64012 | 2 |

-----------
